# Supplementary material for: An EPR Study on Highly Stable Nitroxyl-Nitroxyl Biradicals for Dynamic Nuclear Polarization Applications at High Magnetic Fields
Source: Molecules. 2023 Feb 17;28(4):1926. doi: 10.3390/molecules28041926 (PMC9958542; doi:10.3390/molecules28041926)
Supplement: Supplementary file 1 [file molecules-28-01926-s001.zip › molecules-2226686-supplementary.pdf]

Supplementary Materials

An EPR Study on Highly Stable Nitroxyl-Nitroxyl Biradicals for Dynamic Nuclear Polarization Applications at High Magnetic Fields

Nargiz B. Asanbaeva [1], Sergey A. Dobrynin [1], Denis A. Morozov [1], Nadia Haro-Mares [2], Torsten Gutmann [2], Gerd Buntkowsky [2] and Elena G. Bagryanskaya [1,*]

[1] N.N. Vorozhtsov Institute of Organic Chemistry, 9 Ac. Lavrentiev Avenue, Novosibirsk 630090, Russia
[2] TU Darmstadt, Eduard-Zintl-Institute for Inorganic and Physical Chemistry, Alarich-Weiss-Straße 8, 64287 Darmstadt, Germany
* Correspondence: egbagryanskaya@nioch.nsc.ru

1. EPR sample preparation

EPR samples for W-band measurements of 3–4 were dissolved at a concentration of ~100 μM in a mixture of deuterated DMSO and methanol (at 20:30, v/v) immediately before the experiments. Approximately 2 μL aliquots of these solutions were placed into quartz W-band EPR tubes (0.6 mm inner diameter, open at one end), which were shock-frozen in liquid nitrogen prior to insertion into the precooled cavity. The solvent mixtures used resulted in transparent glassy samples upon rapid freezing.

2. W-band echo-detected spectra

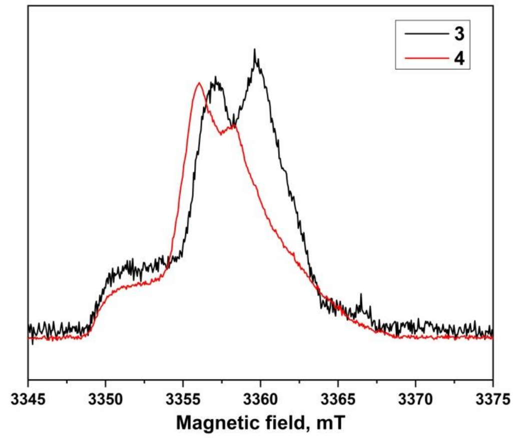

Figure S1. 94 GHz echo-detected EPR spectra (black – for **3**, red – for **4**) at 80 K.

Pulsed EPR experiments at W-band (94 GHz) microwave (MW) frequency were acquired on a Bruker Elexsys E680X EPR spectrometer. π/2 pulse lengths of 16 ns were achieved. The experiments were carried out at a temperature of 80 K.

3. Relaxation measurements

The spin-lattice relaxation time of the biradicals was measured by the inversion recovery method. The phase relaxation time of the samples was measured by means of primary echo decay.

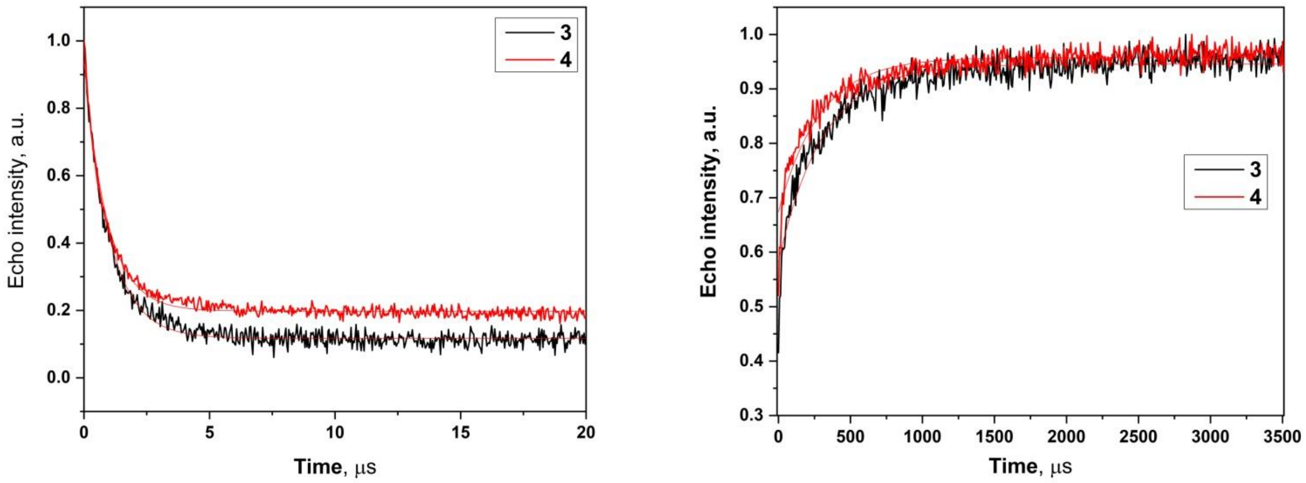

Figure S2. Left: Intensity of spin echo of biradicals **3-4** at 80 K as a function of the time. **Right:** Inversion-recovery time traces of biradicals **3-4** at 80 K along with mono-exponential fits.

The obtained traces were fitted by mono-exponential functions. The results of fitting are presented in Table S1.

Table S1. Parameters of relaxation times for biradicals **3-4.**

**Biradical**	**T_2, μs**	**T_1, ms**
**3**	1.01 ± 0.01	0.31 ± 0.01
**4**	0.92 ± 0.01	0.29 ± 0.01
